# The Utility of High-Resolution Melting Analysis of SNP Nucleated PCR Amplicons—An MLST Based *Staphylococcus aureus* Typing Scheme

**DOI:** 10.1371/journal.pone.0019749

**Published:** 2011-06-22

**Authors:** Rachael A. Lilliebridge, Steven Y.C. Tong, Philip M. Giffard, Deborah C. Holt

**Affiliations:** Tropical and Emerging Infectious Diseases Division, Menzies School of Health Research, Charles Darwin University, Darwin, Northern Territory, Australia; National Institutes of Health, United States of America

## Abstract

High resolution melting (HRM) analysis is gaining prominence as a method for discriminating DNA sequence variants. Its advantage is that it is performed in a real-time PCR device, and the PCR amplification and HRM analysis are closed tube, and effectively single step. We have developed an HRM-based method for *Staphylococcus aureus* genotyping. Eight single nucleotide polymorphisms (SNPs) were derived from the *S. aureus* multi-locus sequence typing (MLST) database on the basis of maximized Simpson's Index of Diversity. Only G↔A, G↔T, C↔A, C↔T SNPs were considered for inclusion, to facilitate allele discrimination by HRM. *In silico* experiments revealed that DNA fragments incorporating the SNPs give much higher resolving power than randomly selected fragments. It was shown that the predicted optimum fragment size for HRM analysis was 200 bp, and that other SNPs within the fragments contribute to the resolving power. Six DNA fragments ranging from 83 bp to 219 bp, incorporating the resolution optimized SNPs were designed. HRM analysis of these fragments using 94 diverse *S. aureus* isolates of known sequence type or clonal complex (CC) revealed that sequence variants are resolved largely in accordance with G+C content. A combination of experimental results and *in silico* prediction indicates that HRM analysis resolves *S. aureus* into 268 “melt types” (MelTs), and provides a Simpson's Index of Diversity of 0.978 with respect to MLST. There is a high concordance between HRM analysis and the MLST defined CCs. We have generated a Microsoft Excel key which facilitates data interpretation and translation between MelT and MLST data. The potential of this approach for genotyping other bacterial pathogens was investigated using a computerized approach to estimate the densities of SNPs with unlinked allelic states. The MLST databases for all species tested contained abundant unlinked SNPs, thus suggesting that high resolving power is not dependent upon large numbers of SNPs.

## Introduction

The identification and monitoring of strains of pathogenic bacteria by genotyping is central to public health, infection control, and veterinary and food microbiology. Electrophoretic techniques such as pulsed-field gel electrophoresis and amplified fragment length polymorphism have been used extensively for many years. More recently, methods that involve the interrogation of known polymorphic regions, such as multi-locus variable number tandem repeat analysis and multilocus sequence typing (MLST), have become prominent. Rapid advances in whole genome sequencing using “next generation” technology means that time consuming and/or expensive genotyping methods will likely become non-competitive in the short to medium term. However, methods based on single-nucleotide polymorphisms (SNPs) will likely remain attractive due to the many efficient and adaptable methods for SNP interrogation, the increasing amount of publicly available comparative sequence data, and increasing understanding of the informative power of SNPs and SNP combinations.

A previously described approach to SNP-based bacterial genotyping involved the computerized derivation from sequence alignments of sets of SNPs that are optimized with respect to the Simpson's Index of Diversity (*D*), and the interrogation of these SNPs using allele specific real-time PCR (kinetic PCR). In this context, *D* is the probability that two sequences in the alignment, selected at random without replacement, will be discriminated by the SNPs. This approach has been applied to several species, and in general has been based upon SNPs derived from MLST datasets [1], [2], [3], [4], [5].

In the case of *Staphylococcus aureus* it was found that eight SNPs divided the MLST database into 47 genotypes that are concordant with the major clonal complexes (CCs) [3]. In a completely clonal population structure, the number of genotypes defined by SNPs will equal the number of SNPs+1 [3], [5], [6], [7], assuming the SNPs are bi-allelic. It was inferred that an excess of genotypes over SNPs is a consequence of horizontal gene transfer (HGT), while concordance between SNP genotypes and the population structure is a consequence of HGT being quite rare, and the CCs reflecting actual biological entities that are genetically isolated from each other to a significant extent.

Recently, a variant of this approach has been described, in which DNA fragments encompassing the resolution-optimized SNPs are interrogated by high-resolution melting (HRM) analysis [8]. The fragments may also include other SNPs that increase the resolving power. The method is “single-step, closed tube”, and requires only a real-time PCR device, generic master-mix and unlabelled primers. This has been termed “SNP nucleated mini-MLST typing”, which we abbreviate to “Minim typing”. Integral to the method are bioinformatic tools that allow facile translation between MLST and Minim data [8].

Here we describe a method for Minim typing of *S. aureus*, and also explore the properties of the Minim typing approach and it's potential to be applied to other bacterial species. This study showed that Minim typing subdivides *S. aureus* in a manner concordant with its population structure. It also revealed that prominent species of bacterial pathogens contain large numbers of SNPs that have been subjected to HGT and so have the potential to underpin highly discriminatory genotyping methods. As larger datasets of whole genome data become available this approach will facilitate an optimized but parsimonious use of genome wide SNPs.

## Results

To investigate the properties of the Minim typing approach we began by determining that fragment sizes of 150–200 bp represented a good compromise between sequence diversity, and the ability to reliably resolve distinct. We then identified a set of SNPs optimised for resolving *S. aureus* strains from each other, and designed HRM fragments of the appropriate size that contained these SNPs. The high resolving power of the six chosen fragments in relation to MLST was confirmed by comparing the chosen fragments with random fragments of the same size and also demonstrating that the fragments covered regions of the concatenated MLST sequence that are associated with a high Simpson's Index of Diversity. Following these *in silico* analyzes, we tested the method on 95 clinical *S. aureus* isolates and found that the HRM curves were reproducible and the order of curves essentially as predicted with minor exceptions. We generated a key that facilitates translation between HRM based genotypes and MLST and demonstrated that the clustering of HRM genotypes is concordant with the *S. aureus* population structure as defined by MLST.

### Prediction of effective fragment sizes for Minim typing

The predicted effective fragment sizes for Minim typing were calculated. We conservatively assumed that sequence variations that do not affect the G+C content are not revealed by HRM, even though this is not always the case. First, we addressed the question as to the upper limit on the size of fragments for which sequence variants may be reliably discriminated by HRM analysis. Our experience with the Corbett Rotorgene is that the limit for the reliable detection of differences in melting temperatures (ΔT_m_) is 0.2°C. The predicted ΔT_m_ caused by a single change in the number of G or C residues decreases as the length of the fragment increases. Assuming a constant ionic environment, this is described by ΔT_m_ = 41((GC_2_–GC_1_)/N), where GC is the number of G+C residues and N is the length of the fragments [9], [10]. According to this equation, the ΔT_m_ conferred by a single change in the number of G or C residues in a 200 bp fragment is approximately 0.2°C. It was concluded that variants of fragments >200 bp may not always be discriminated by HRM analysis, even when they differ in G+C content.

**Figure 1:**
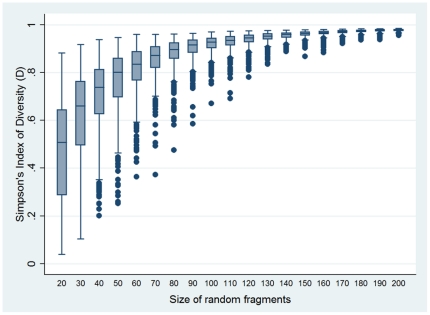
The resolution obtained with increasing coverage of the concatenated MLST sequence. The resolution obtained was calculated by generating six random fragments 20 bp to 200 bp in 10 bp increments, with 500 iterations.

Second, we performed an *in silico* examination of the relationship between fragment size and resolving power, assuming all variations in G+C content are detected. Six randomly selected fragments, each of the same length, were derived from the aligned concatenated *S. aureus* MLST database, and *D* value for the combined six fragments calculated by assigning alleles to each fragment based on G+C content. This was repeated 500 times each for fragment sizes ranging from 20 to 200 bp in increments of 10 bp. There was a clear correlation between resolving power and fragment size that did not flatten before reaching 150–200 bp (Figure 1). It was therefore concluded that there is no benefit in using fragments smaller than 150–200 bp for reliable HRM analysis.

### Identification of HRM informative regions in the *S. aureus* MLST data

Unidirectional nucleotide pair frequency calculations of the concatenated *S. aureus* MLST dataset revealed that each ST differed from any other ST at an average of 27 nucleotides and that 22 (81%) of these involved a change in G+C content that would likely facilitate allele discrimination by HRM analysis. To design the Minim typing method, the program Minimum SNPs was used to identify highly informative Simpson's Index of Diversity (*D*) optimized SNPs that change the G+C content. Six fragments were identified that encompassed SNPs at positions 78 and 210 of the *arcC* locus, positions 88 and 155 of the *aroE* locus, position 286 of the *gmk* locus, position 294 of the *pta* locus, position 36, and positions 241 and 243 of the *tpi* locus. There were considerable constraints on primer design, and as result, even though the aim was for the fragments to be close to 200 bp, they ranged in size from 83 bp to 219 bp (Table 1). Each ST was assigned a predicted MelT profile. The six Minim fragments define 275 predicted MelT profiles, and provide a *D* of 0.979 compared to full MLST for the 1420 STs included in the analysis.

**Table 1:** Details of the six fragments used in the *S. aureus* typing scheme.

Fragment name	SNP(s) position(s) in concatentated MLST sequence	Region interrogated by HRM	Primers (5′-3′)	Fragment size (bp)	HRM normalisation regions (°C)	Number of predicted curves	Predicted Δ Tm (°C)
*arcC78/210*	78, 210	73–210	TGGATACTTGTGGTGCAATG	181	74–75	6	0.23
			CGTATAAAAAGGACCAATTGGTTT		80–81		
*aroE88/155*	543, 610	521–617	TAAATATTCCAATTGAAGATTTTC	140	67–68	6	0.29
			CTGCATTAATCGCTTGTTCA		76–77		
*gmk286*	1663	1637–1680	GAAGTAGAAGGTGCAAAGC	83	69–70	4	0.49
			CAAGTGATCTAAACTTGGAGG		77–78		
*pta294*	2100	2100–2216	TGCAGCACATTCAACAGG	158	74–75	5	0.26
			CCTTGTGAATCAAGTTCTGGATTG		82–83		
*tpi36*	2316	2316–2485	GATGAAGAAATTAACAAAAAAGCGCA	219	74–75	6	0.19
			TTGATGATTTACCAGTTCCGATTG		81–82		
*tpi241/243*	2521, 2523	2521–2644	GTAAATCATCAACATCTGAAGAT	168	73–74	4	0.24
			GCCCCATCAATATCAGTTTGTG		79–80		

The normalization regions refer to the temperatures selected to normalize the florescence curves using the Corbett Rotorgene software.

### The SNP nucleated fragments are more discriminatory than randomly selected fragments

**Figure 2:**
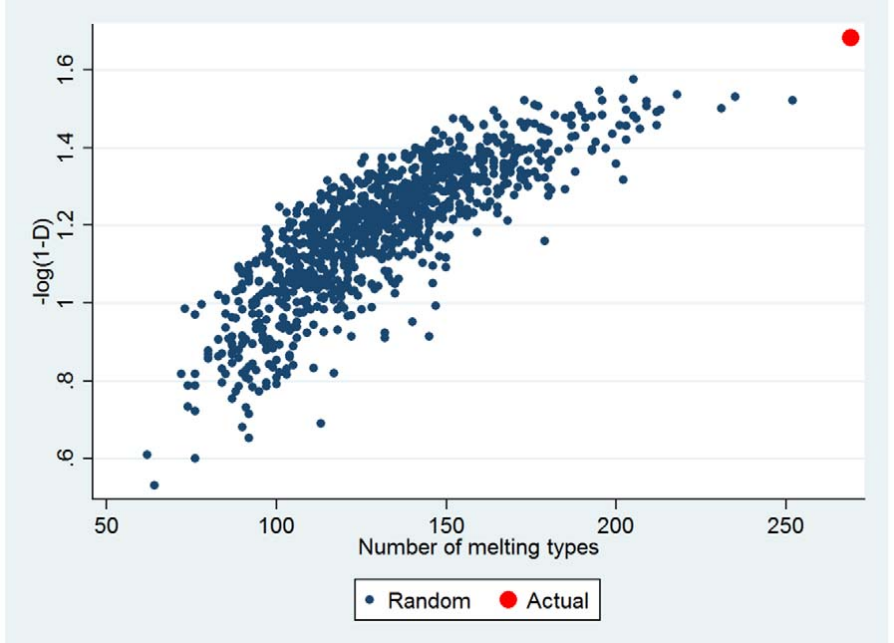
Comparison of the resolution obtained with the actual selected fragments and 1000 randomly chosen fragments. The actual fragments outperforms randomly selected fragments with the generation of more melting types and a larger*–log(1-D)* value.

Two different approaches independent of the Minimum SNPs-based SNP selection procedure were used to investigate the extent that the selected Minim fragments are resolution optimized. First, we ran a simulation to randomly select 6 fragments, each of which was equal in size to one of our 6 selected fragments. After 1000 simulations, the mean number of MelT profiles was 131 (SD 28) and the mean–log(1-D) value was 1.19 (SD 0.17), with none of the randomly selected combinations providing better resolution than the fragments we originally selected that provided 275 MelT profiles and a–log(1-D) of 1.68 (Figure 2).

**Figure 3:**
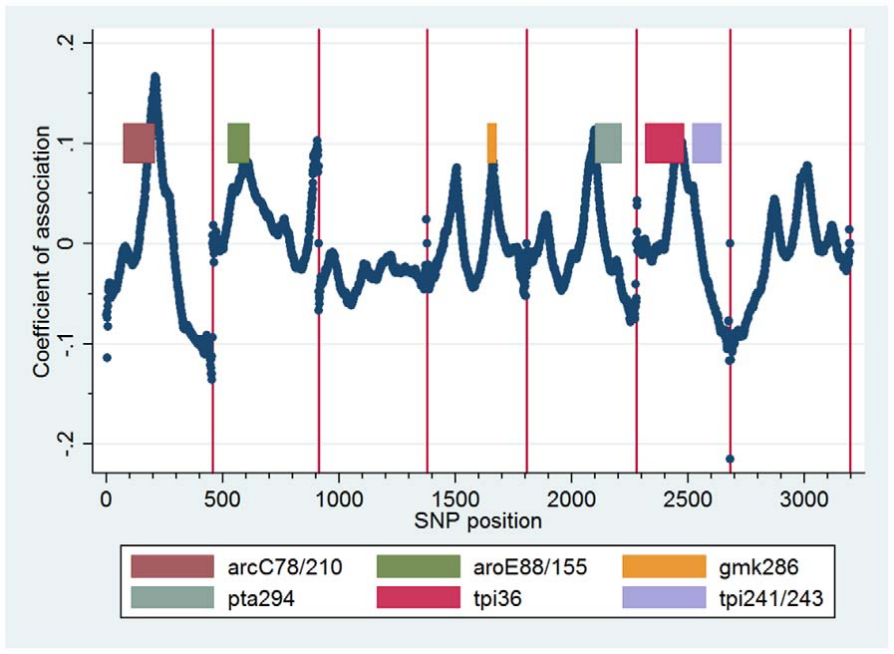
SNP association map for *S. aureus* showing the positions of the six selected fragments. Peaks in the coefficient of association demonstrate regions which, if included in random fragments, are associated with a high *–log(1-D)* value (i.e., contain informative SNPs). This analysis confirms that the fragments selected using Minimum SNPs and HRMtype include highly informative SNPs.

Second, we investigated the association between increasing resolution and incorporation of individual SNPs in the randomly selected regions by generating a SNP association map. This demonstrated that there are discrete regions of the concatenated MLST sequence that, if included in the selected regions, independently result in high *D* values (Figure 3). These regions correlate with the *D* optimized SNPs chosen by Minimum SNPs and the reason our 6 selected regions perform so well is that the majority of these independent regions are included. Thus this SNP association map confirms the utility of Minimum SNPs but also provides a graphical means of representing the number and relative importance of *D* optimized SNPs across the whole concatenated sequence.

### Method Validation

The Minim method was tested using a reference collection of 93 clinical isolates from the Royal Darwin Hospital that had been assigned to clonal complexes by a SNP-based kinetic PCR technique and of which a limited number also had full MLST data [11]. Our collection includes the major clonal complexes present in Australia apart from ST59. We therefore also included a ST59 isolate from Western Australia [12]. Together, the CCs of the isolates include CC1, 5, 8, 15, 20, 22, 25, 30, 45, 59, 88, 93, 97, 104, 121, 188, 239, 779. We used a ST239 isolate as a control for each fragment as it is a commonly found ST and its predicted curves are close to the middle of the predicted range of T_m_ values, thus facilitating assignment of other curves relative to the ST239 curve. The collection was predicted to encompass 4 of the 6 predicted curves for *arcC78/210*, 4 of 6 for *aroE88/155,* 3 of 4 for *gmk286*, 3 of 5 for *pta294*, 5 of 6 for *tpi36* and 3 of 4 for *tpi241/243*.

**Figure 4:**
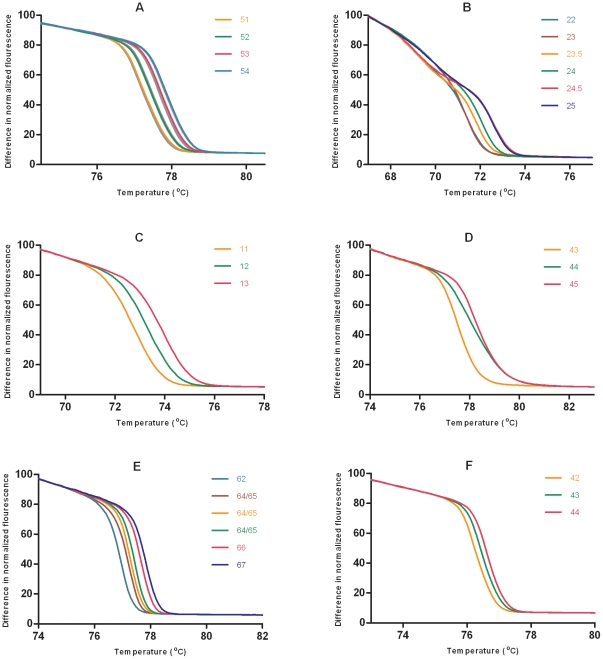
High-resolution melting curves for the six fragments. The six fragments are (A) *arcC78/210*, (B) *aroE88/155*, (C) *gmk286*, (D) *pta294*, (E) *tpi36* and (F) t*pi241/243*. The curves are labeled by the number of G+C residues contained in the corresponding fragment. For *arcC78/210* a total of 24 curves are presented to demonstrate the reproducibility and ability to discriminate multiple curves (replicate curves colored grey). All other regions are presented with one representative of each curve present in the isolate collection.

**Figure 5:**
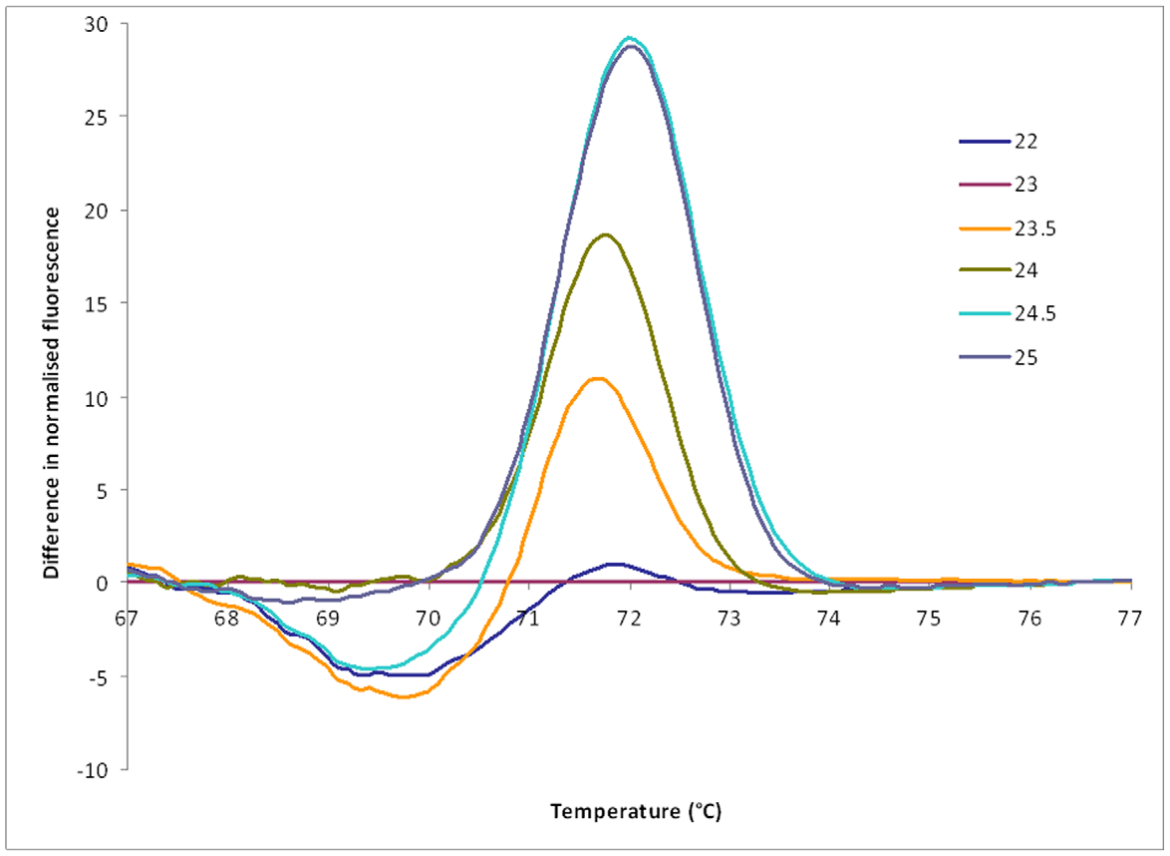
Difference graph for *aroE88/155*. The high-resolution melting curves are compared with curve 23 (represented by ST239) as the baseline. The two melting domains are evident with the first between 68 and 71°C and the second between 71 and 74°C.

The HRM curves produced were reproducible, and the order and ΔT_m_ values of the curves were essentially as predicted (Figure 4). To provide an indication of the reproducibility, for 122 tested replicate reactions from across the six fragments, the mean pairwise difference in T_m_ was 0.03°C (SD 0.02). The HRM curves can also be represented as difference graphs by using one of the curves as a baseline comparator. For the *aroE88/155* fragment in particular, the difference graph using the ST239 control (curve 23) as the baseline, better demonstrated the separation between curves (Figure 5). Exceptions to the predicted curves were present in *aroE88/155*, *tpi*36 and *tpi241/243*. It was evident that the *aroE88/155* fragment melted in two domains at 69–71°C and at 71–74°C. This resulted in differentiation of some sequence variants with the same G+C content, thus providing better than expected resolution. We assigned the new *aroE88/155* curves as 23.5 and 24.5 (Figure 4*B*). For *tpi36* there was an additional curve consistently produced by ST93 isolates that had a higher T_m_ than predicted. We assigned this new *tpi36* curve as 67 as its ΔT_m_ from curve 66 was approximately that of the predicted curve 67, although we had no representatives of curve 67 in our collection to verify this (Figure 4*E*). We were not able to clearly differentiate predicted *tpi36* curves 64 and 65 when a large number of isolates were analyzed and so have conservatively placed them together as curve 64/65. Finally, for *tpi241/243*, ST59 produced a lower T_m_ than predicted and its curve was reassigned from 43 to 42; and ST93 was predicted to produce curve 44 but produced a T_m_ between that of curve 44 and curve 43 that could not always be distinguished from curve 43. In the context of using the other five loci where ST93 has a unique profile this does not result in any confusion in distinguishing ST93. Sequence determination demonstrated complete concordance between sequence and these new HRM curves. Following these re-assignments the method provides a total of 268 MelT profiles and a *D* value of 0.978 (95% confidence interval 0.976–0.981) with respect to the 1420 MLST sequence types. A Microsoft Excel “MelT key” that facilitates Minim data interpretation and translation between MelT and MLST data was assembled (Data S1). This encompasses the information regarding the unexpected HRM curves. A suggested protocol for applying the Minim technique and use of the MelT key is provided in Text S1.

The results obtained with the Minim typing method largely correlated with the results previously obtained using the kinetic PCR SNP typing method. For isolates where a discrepancy occurred between the methods, the relevant MLST locus of the isolate was sequenced. In all cases the Minim typing result was correct, with the kinetic PCR SNP result failing to resolve single locus variants within clonal complexes (Data S2). A comparison of Minim typing with the kinetic PCR SNP method for the 1420 MLST sequence types confirmed the concordance between the methods with the Wallace's coefficient for (MelT→SNP) = 0.90 (95% CI 0.87–0.92) and Wallace (SNP→MelT) = 0.34 (95% CI 0.31–0.37). This indicates that if two strains are clustered together by MelT, there is a 90% probability they will be clustered together by the SNP method; however, due to the lower resolution of the SNP method, there is only a 34% probability that two strains clustered together by the SNP method will be clustered together by MelT.

On the basis of the criteria we have developed for discriminating HRM curves, on no occasion did replicate analysis of the same isolate yield discrepant results. This indicates a reproducibility of 100%. This was determined from eight isolates that were completely analysed more than once, with the total number of analyses being 38 (i.e a mean of 4.75 replicates per isolate). Additional replicate analyses of these isolates were carried out at subsets of the loci. The total number of individual HRM assays carried out was 640, which may be regarded as equivalent to 107 complete Minim typing procedures, with a mean of 13.4 replicates per isolate. Another indication of robustness was that 155 HRM analyses, spread approximately equally across the loci, were performed on known sequences. The HRM data was all in accordance with the sequence data.

### The clustering of HRM genotypes is concordant with the *S. aureus* population structure as defined by MLST

It is desirable that *S. aureus* Minim typing be concordant with the population structure defined by MLST. In particular, this would allow the inference of the CC from the MelT data. We tested this by two means. First, we calculated Wallace's coefficient to compare MelT with clonal complexes and sub-clonal complexes (subCC) as defined by eBURST analysis for the 1420 STs, including 201 singletons. This yielded Wallace (MelT → CC) = 0.85 (95% CI 0.82–0.88) and Wallace (MelT→subCC) = 0.73 (95% CI 0.69–0.77). This indicates that if two strains are in the same cluster by MelT they have an 85% chance of having the same CC and 73% chance of having the same subCC. This analysis assumes an equal abundance of all STs, so the calculations were repeated using MLST data that better reflects the differing abundances of STs. In the MLST database, 3318 submitted isolates had STs that were present in the 1420 STs used to generate the MelT key. The ST data from these yielded Wallace (MelT→CC) = 0.949 (95% CI 0.938–0.961) and Wallace (MelT→subCC) = 0.841 (95% CI 0.0.823–0.860). These higher values reflect the abundance in the database of isolates with CC and subCC founder STs, and the effectiveness of Minim typing at discriminating these founder STs. The discriminatory power of Minim typing with this data set of 3318 isolates is *D* = 0.961 (95% CI 0.959–0.963), which is comparable to that of MLST at *D* = 0.987 (95% CI 0.983–0.989). The subCC data using MLST provides *D* = 0.957, which is less than that provided by Minim typing. Therefore Minim typing is concordant with MLST and provides discriminatory power significantly beyond assignment into CCs and subCCs.

Second, the STs defined by MelTs were examined individually. In general, for CCs of significant size, a single MelT encompasses a CC founder and large proportion of its SLVs, and this MelT does not encompass the founder of any other CCs. Many MelTs encompass no major CC founder, but rather encompass a small number of closely related STs, such as SLVs of the same CC founder. These findings may be illustrated using the large and diverse CC8. The sensitivity, specificity and positive predictive values (PPV) of Minim typing for CCs and subCCs were calculated, using the simplifying assumption that all STs are equally abundant (Table 2). MelTs diagnostic for a CC or subCC were defined as MelTs for which at least 50% of the STs belong to the CC or subCC of interest. False positives were STs that are encompassed by a diagnostic MelT and not belonging to the CC or subCC of interest. False negatives were STs belonging to the CC or subCC of interest, but defined by MelTs that were not diagnostic for the CC or subCC of interest. The specificities were universally high, and this is primarily a function of the high numbers of true negatives (Table 2). Sensitivities and PPVs were also high, but more variable, primarily because of the smaller and more variable number of true positives, with the PPVs for the small subCCs being depressed by small absolute numbers of false positives. If singletons that are DLVs of a CC founder are assigned to that CC, and STs that are SLVs of two founders are assigned to the CC that provides the “best” diagnostic parameters, then the enhanced parameters show large improvements, particularly for the PPVs (Table 2). This is primarily due to the reclassification of false positives as true positives. The generation of new false negatives that are singleton DLVs of CC/subCCs of interest, and not within MelTs diagnostic for the CC/subCCs of interest, had only a very small effect. The only significant anomaly brought into focus by this analysis was that the founders of CC7 and the subCC8-247 are not discriminated.

**Table 2:** Diagnostic parameters of Minim typing for CC8 and subCCs within CC8.

CC/subCC	STs^a^	MelTs^b^	Sensitivity		Specificity		PPV	
			Raw^c^	Enhanced^d^	Raw	Enhanced	Raw	Enhanced
8 (all)	185	22	0.973	0.965	0.976	0.983	0.863	0.906
8–8	110	10	0.891	0.935	0.992	0.998	0.899	0.972
8–239	44	7	0.911	0.948	0.992	0.997	0.788	0.932
8–72	14	1	0.717	0.733	0.996	0.996	0.630	0.688
8–247	11	0^e^	ND	ND	ND	ND	ND	ND
8–770	6	2	0.830	0.897	0.997	0.997	0.560	0.625

The calculations assumed equal abundance of all STs. ND = not determined.

a Number of STs belonging to the CC/subCC.

b Number of MelTs diagnostic for the CC/subCC. Each MelT defines either a single ST belonging to the CC/subCC of interest, or a group of STs of which at least 50% are of the CC/subCC of interest.

c The CC and subCC assignments for STs in the Excel key were used without modification.

d Singletons that are DLVs of the founder of the CC/subCC were classified as true positives, and false positive and false negative STs that are SLVs for more than one CC/subCC founder were assigned to the CC/subCC that maximizes the diagnostic parameters. Additional singletons that are founder DLVs and do not correspond to MelT diagnostic for the CC/subCC of interest were identified as new false negatives.

e There are no MelTs diagnostic for subCC 8–247 according to the criterion used, primarily because CC7 and subCC 8–247 are not discriminated by Minim typing.

### The potential resolving powers of SNP typing in other bacterial species

The value of the Minim approach is in part a function of the density of informative SNPs. If a sequence alignment can be converted to a completely consistent dendrogram, then there is no homoplasy, no evidence for recombination involving the SNPs and the resolving power of SNPs defined by the sequence variants can only increase arithmetically as SNPs are added to the typing set. Conversely, if recombination is extensive, the resolving power can increase exponentially as SNPs are added.

At any point in the assembly of a set of resolution optimized SNPs, a pool of SNPs is available for selection as the next SNP to be added to the set. It was reasoned that the absolute number of such SNPs that individually provide a much greater increase in resolving power than is possible for an unrecombined SNP, is a useful measure of the density of recombined SNPs that confer considerable resolving power when used for genotyping. To determine the densities of such SNPs in different bacterial pathogens, an *in silico* “SNP depletion” experiment was carried out using the different MLST datasets. Highly resolving SNPs were least dense in *S. aureus*, confirming a previous analysis [13], while *Haemophilus influenzae* and *Streptococcus pneumoniae* have particularly high densities of informative SNPs (Table 3).

**Table 3:** Densities of highly recombined SNPs in seven species.

MLST dataset	Resolution provided by first five SNPs in the set (*D*)	SNPs that at position six in the set confer >5X maximum resolution increase for non-recombined SNPs.	Size of MLST dataset (bp)	SNPs per kb	Average interval between SNPs (bp)
*S. aureus*	0.926	0^a^	3198	<0.31	>3198
*S. pyogenes*	0.962	21	3134	6.7	149
*C. jejuni/coli*	0.945	2	3309	0.6	1655
*B. pseudomallei*	0.949	9	3456	2.6	384
*S. pneumoniae*	0.963	40	2751	15.5	69
*E. faecium*	0.943	6	3458	1.73	576
*H. influenzae*	0.983	>75^b^	3057	>24.5	<41

a The largest increase provided by any SNP at position six was 3.45x the maximum possible by a non-recombined SNP.

b The depletion experiment was terminated after 75 SNPs had been excluded from analysis. The final SNP tested provided an increase in resolving power 8.38x the maximum possible by a non-recombined SNP.

## Discussion

Despite the increasing capability to rapidly generate sequencing data at multilocus and genome wide levels, genetic analysis methods that are inherently rapid and simple, and that make use of generic equipment and reagents, are likely to remain useful for some time. The large volume of comparative sequence data on the internet provides an excellent resource for the identification of highly informative subsets of known polymorphic sites or regions that may be used in such methods. We demonstrate the practical application of such an approach to genotyping *S. aureus* by identifying resolution optimized SNP combinations in MLST datasets and interrogating these SNPs with the low cost HRM platform. Six single-step PCR and HRM reactions were shown to provide a high degree of resolution compared to full MLST with a *D* value of 0.978.

There are several limitations to this Minim typing approach. First, given the available HRM technology, it is not usually possible to discriminate fragments with the same G+C content but of a different sequence. Therefore, in generating the translation key we conservatively assumed that fragments with the same G+C content will produce the same curve. As a consequence, the discriminatory power of HRM typing will always be less than that of full MLST. Also, new MLST alleles that have the same G+C content as previous alleles in the interrogated fragment will not be revealed through Minim typing. Although it would have been ideal to have had full MLST sequences for all tested isolates, to reduce the amount of sequencing required, we made initial comparison with the kinetic SNP PCR typing method and any discrepancies with Minim typing were resolved by sequencing of the relevant MLST alleles. Finally, as the MLST database is updated with new STs and CCs being deposited, the translation key will concordantly need to be updated, therefore we have established a website where updated keys can be accessed (www.menzies.edu.au/research/tropical-and-emerging-infectious-disease/bacterial-genotyping). In addition, there is much that is arbitrary about MLST itself. The choice of different numbers of fragments, sizes of fragments, or genes used for MLST would have resulted in many differences in detail concerning which isolates are resolved by MLST and which are not–but this would not have affected greatly the general model of the population structure, and the assignment of isolates to particular lineages. Exact concordance with MLST typing has little or no biological meaning.

Nonetheless, HRM analysis was robust. There were no instances of the same alleles being falsely identified as different, while the HRM curves from fragments of differing G+C content were identified as different with one exception where two sets of curves could not be confidently discriminated. There were several instances where different alleles with the same G+C content were resolved. The key for converting MelTs to STs was easily adjusted to incorporate this additional information. The relationship between fragment size and the T_m_ difference conferred by a single change in G+C content suggests we have used fragments close to the optimum for HRM analysis, with smaller fragments providing fewer alleles, and alleles of fragments larger than 200 bp being potentially difficult to resolve.

There is considerable concordance between *S. aureus* MLST and Minim typing. Disjunction between the MLST population structure and the MelTs arises from two mechanisms. First, many are consequences of limitations of the eBURST algorithm or convention by which we have defined CC's. For example, singletons that are encompassed by a MelT strongly specific for a particular CC are very often DLVs of the founder of that CC. Also, very large CCs often encompass STs that are not closely related at all, but are included in the same CC simply because intensive sampling of the population has resulted in a network of STs, all connected by SLV relationships. This can result in STs assigned to the same CC being less closely related to each other than to STs assigned to different CCs. Second, there are instances in which genuinely unrelated STs are not discriminated. These are rare, and also the MelTs for which this is the case can be readily identified using the Excel key. If unambiguous assignment to an MLST-defined CC is necessary, then ambiguous MelTs may trigger further analysis. However for many purposes, defining the isolate simply on the basis of its MelT, and accepting occasional and minor ambiguity in precise assignment to CCs, would be sufficient.

The Minim approach can be applied to other important bacterial species and we provide the theoretical basis for choosing SNPs and indications as to the resolving power achievable for several different species. Determination of the density of highly informative SNPs in different species showed that such SNPs can be very abundant, with *H. influenzae* and *S. pneumoniae* being conspicuous examples. This indicates that any genotyping technology that interrogates significant numbers of these SNPs in these species will provide very high resolving power. It is likely that the correlation between MelT types and population structure will be inferior in highly diverse and recombined populations. However this would represent the difficulty of depicting the complete phylogeny of strains in a recombining population and the limitations of eBURST and similar algorithms, rather than any fundamental failing of the Minim approach. The basis for differences between the densities of recombined SNPs in the different bacterial species is almost certainly a function of SNP diversity (i.e., the total density of SNPs, which will be a function of the antiquity of the species) and the speed with which the SNP is recombined throughout the population, which will be a function of rate of HGT. *B. pseudomallei* is notable because its SNP diversity is low, so the pool of informative SNPs is shallow, but the most informative SNPs provide very good resolving power. This suggests a high rate of HGT, which indeed has been previously reported [13]. Our analysis suggests that *Streptococcus pyogenes* has a similar population structure. The results from *C. jejuni*/*C. coli* were of interest because they showed a low density of recombined SNPs, which appears inconsistent with the reported high frequency of recombination [13]. This is likely due to the incorporation of two species in the MLST database, which means that SNPs provide resolving power in one species or the other but not both, thus reducing the potential resolving of the SNPs with reference to the MLST database.

The use of HRMType allows the accurate *in-silico* generation and optimization of the typing scheme. The protocol we have developed for identifying regions optimized for genotyping based upon G+C content and interpreting the data with reference to databases of sequence variation could easily be adapted to select fragments and generate a key for other platforms such as with base composition analysis using PCR/ESI-MS [14]. The method is also flexible in that the number, size and position of the fragments can be varied and therefore a typing scheme can be tailored according to the needs of the user, balancing convenience, resolving power and cost.

HRM based typing has several attractive features including that the amplification and HRM analysis is a single-step, closed tube process. The only manipulations required are DNA extraction and PCR reaction set-up, and the cost is minimal with only six PCR reactions using generic real-time PCR master-mix. It is equally cost effective as a high-throughput method or for small numbers of samples and allows for easy inter-laboratory comparison of results. Our typing scheme for *S. aureus* could usefully be incorporated into a progressive hierarchical typing scheme [6]. For *S. aureus* this could be used in conjunction with interrogation of hypervariable regions (e.g., *spa* typing [15], [16]) and virulence and resistance genes, all of which could be performed on the same technology platform.

## Materials and Methods

### Concatenated *S. aureus* MLST sequences

The concatenated MLST sequences for 1444 STs (ie up to ST1508) were downloaded from the *S. aureus* MLST web site (saueus.mlst.net) in November 2009. Four STs (STs 753, 957, 1166 and 1471) with an incorrect concatenated sequence length and one ST (ST1110) with a likely frame shift at position 81 of the *glpF* locus were subsequently removed from the alignment. Ten STs (STs 102, 202, 204, 208, 215, 220, 663, 723, 1079, 1407) were removed on the basis that their *tpi* alleles and in some cases *pta* alleles were >13% diverged from the other *S. aureus* alleles and all but one had a best BLASTn hit to a Staphylococcus species other than *S. aureus*. These alleles would likely fail to amplify or amplify late during PCR using the primers we designed using the *S. aureus* allele sequences, due to polymorphisms in the primer binding regions. These STs were appended to the MelT key (see below) with a note indicating that one or more of the regions may fail to amplify, and thus they will not produce a complete MelT profile. A further nine STs which are likely members of clonal complex 75 (STs 75, 258, 850, 883, 1223, 1284, 1288, 1303, and 1304) were also removed as this clonal complex shows significant divergence from other *S. aureus* and may represent a distinct species or sub-species [17]. The remaining 1420 concatenated sequences were analyzed using MEGA 4.02 [18].

### Identification of D optimized SNPs useful for HRM analysis

We had previously identified a set of eight SNPs with a Simpsons Index of Diversity (*D*) of 0.95 for the MLST dataset [3]. We now aimed to generate a set of *D* optimized SNPs which were likely to contribute to a change in the melting temperature of the DNA (i.e., principally transitions) for interrogation by HRM. From the original SNP set, we used SNPs which were transitions for most STs (positions 210, 2100, 2316 and the 2521/2523 combination) as forced inclusions and SNPs which were transversions (positions 162, 1695 and 2189) as forced exclusions in Minimum SNPs [1], [19]. Any additional SNPs identified by Minimum SNPs which were transversions for most STs were further excluded. Transitions which were in close proximity to each other were favored as they could be included in a single small PCR product for HRM analysis. Primers were manually designed in the regions flanking the identified SNPs in each locus to give products ranging in size from 83–219 bp (Table 1).

### HRM curve prediction and assignment of “Melting Type”

We wrote in-house programs as do files in Stata 10.9 (StataCorp, Texas) to analyze regions internal to the primers. We have called the program HRMType and it generates a prediction of the number and order of HRM curves for specified regions from an input sequence alignment (Text S2 and Text S3). For simplicity we assumed that amplicons of a given region would melt in an order based on their G+C content. Each predicted curve was given a number based on its G+C content and an HRM profile was generated for each ST based on the expected curve number for each region. This profile was designated the “Melting Type” (MelT) for the ST and a key was generated which facilitated the translation between MelT profile and ST (Data S1).

### Comparison with randomly generated fragments

We adapted HRMType to calculate the *D* value for randomly generated fragments from the concatenated sequence. Individual fragments were not allowed to span more than one MLST loci in the concatenated sequence. Initially regions with fixed sizes ranging from 20 to 200 bp were used and subsequently regions with the same size as our 6 chosen regions were used. Multiple iterations allowed us to determine the distributions of *D* and the transformation *–log_10_(1-D)* for these randomly generated fragments. Additionally, for each position in the MLST concatenated sequence we performed a multivariate regression analysis with the outcome variable *–log_10_(1-D)* and the independent variable the absence or presence of that position within the selected fragments. Adjustment was made for fragment size. The coefficient from the regression analysis provided a measure of association between each position of the concatenated sequence and the overall resolving power obtained. These coefficients of association were plotted against the concatenated position to generate a SNP association map (Figure 3).

### Isolates collection for validation of methodology

Isolates used for this study were clinical *S. aureus* isolates collected as part of a case-control study at the Royal Darwin Hospital from April 2006 to April 2007 [11]. These isolates had previously been assigned to a clonal complex using a kinetic real time PCR method [20]. A single colony on Horse Blood agar was inoculated into 5 mL of Todd-Hewitt broth (Oxoid) and grown overnight at 37°C with agitation. DNA was extracted from the pelleted cells using a QIAamp DNA mini kit (Qiagen) using the protocol for Gram positive bacteria with lysostaphin. Purified DNA was eluted in 200 ul 10 mM TrisCl, 0.5 mM EDTA, pH9, and diluted 1∶10 in H_2_O prior to use in real time PCR.

### Real time PCR and HRM analysis

Real time PCR reactions contained 5 µL 2x Platinum® SYBR® Green qPCRSuperMix-UDG (Invitrogen Life Technologies), 0.4 uM each primer and 1 ul of the diluted DNA extraction in a total volume of 10 ul. The reactions were performed on a on a Rotorgene 6000 device (Corbett Life Science). Cycling conditions were 50°C for 2 minutes, 95°C for 2 minutes followed by 40 cycles of 95°C for 3 seconds, 56°C for 18 seconds and 72°C for 12 seconds. HRM was carried out from 66°C to 84°C in 0.1°C increments for 2 seconds each. Raw HRM curves were normalized by the Rotorgene 6000 software v 1.7 (Table 1). Difference graphs of the normalized curves were obtained using a ST239 isolate as the baseline curve.

### MLST of specific isolates and loci

Sequencing of selected MLST loci of some isolates was carried out using standard MLST primers and methodology for *S. aureus* [21].

### Determination of the concordance between HRM typing and the MLST-defined *S. aureus* population structure

Wallace's Coefficient was calculated using Comparing Partitions [22]. Determination of the concordance between the predicted MelT and the MLST-defined population structure of *S. aureus* was carried out *in silico*. An eBURST analysis [23] was performed on the entire *S. aureus* MLST database. The group definition was set to “six alleles in common”, and each group was defined as a clonal complex (CC). “SubCCs” within CCs were identified on the basis of the presence of a progenitor with at least five SLVs. In the case of large CCs with several subCCs and chains of STs with SLV relationships, the boundary between sub-CCs was placed at the half way point between sub-CC progenitors on the eBURST diagram. The sensitivity (true positives/(true positives+false negatives)), specificity (true negatives/(true negatives+false positives)) and positive predictive value (true positives/(true positives+false positives)) of MelTs for a CC or sub-CC of interest were calculated.

### Estimation of the density of recombined SNPs in MLST datasets from different bacterial species

For the MLST datasets of *S. aureus, Streptococcus pyogenes, Campylobacter jejuni, Burkeholderia pseudomallei, Streptococcus pneumoniae, Enterococcus faecium* and *Haemophilus influenzae*, the following procedure was followed. Firstly a set of six SNPs that provided an optimized *D* value was derived using the software Minimum SNPs. The derivation was then iterated, with the first five SNPs in the set held constant, and the increase in resolving power provided by the 6^th^ SNP recorded. This 6^th^ SNP was then excluded from analysis in subsequent iterations. This was continued until the increase in resolving power was five times the maximum increase that can be conferred by a non-recombined SNP. Six member SNP sets were used because this provides a comprehensive test of SNP performance, and also yields manageable numbers of alterative SNPs that provide a >5X the maximum increase in resolving power possible for non-recombined SNPs. The “5X” parameter was chosen arbitrarily as representing a figure considerably greater than that possible from a non-recombined SNP.

Normalization was required to determine the maximum increase in resolving power that can be conferred by a non-recombined SNP for each of the different MLST datasets. This is because the different datasets provided different resolving powers from the first five SNPs. Maximum resolving power from recombined SNPs, expressed in terms of number of genotypes, increases logarithmically as SNPs are added to the set. Conversely, maximum resolving power from non-recombined SNPs increases linearly. The log(number of genotypes), assuming all genotypes all contain the same number of STs, is equal to –log(1-*D*). Therefore, the higher the value of –log(1-*D*) conferred by the first five SNPs in the set, the smaller the maximum increase in this parameter that can be conferred by the addition of a non-recombined SNP to the set. This may be represented by an exponential decay function: if *PR* is the resolving power conferred by a set of SNPs, and *MI* is the maximum increase in resolving power that can be conferred by the addition of a non-recombined SNP to the set, then: *MI* = 0.3389e^−2.116*PR*^ (PR and MI are both expressed in terms of –log(1-*D*)). For each of the MLST datasets, this function was used to derive *MI* from the *PR* value conferred by the first five SNPs in the set.

## Supporting Information

Data S1 *Staphylococcus aureus* MelT key.
(XLSX)

Data S2 Comparison of MelT and kinetic PCR SNP typing results.
(XLS)

Text S1 Suggested protocol for typing of clinical isolates.
(DOC)

Text S2 HRMType program.
(TXT)

Text S3 HRMType Manual.
(PDF)
