# Supplementary material for: The Utility of High-Resolution Melting Analysis of SNP Nucleated PCR Amplicons—An MLST Based Staphylococcus aureus Typing Scheme
Source: PLoS One. 2011 Jun 22;6(6):e19749. doi: 10.1371/journal.pone.0019749 (PMC3120814; doi:10.1371/journal.pone.0019749)
Supplement: Text S1 — Suggested protocol for typing of clinical isolates. (DOC) [file pone.0019749.s003.doc]

**Text S1. Suggested protocol for typing of clinical isolates.**

1. Extract DNA using a consistent method so that quantity of DNA, and salt and ionic concentrations are as similar as possible for different isolates.
2. Perform PCR/HRM reactions and include as controls at least one isolate with a known sequence and hence curve (we have used an ST239).
3. Ensure consistent PCR amplification as poor amplification results in inconsistent HRM curves.
4. The HRM curves for each isolate can be assigned a curve number when compared with the known controls.
5. Each isolate is assigned a Melting Type using the translation key based on the combination of curve numbers.
