# Supplementary material for: The Utility of High-Resolution Melting Analysis of SNP Nucleated PCR Amplicons—An MLST Based Staphylococcus aureus Typing Scheme
Source: PLoS One. 2011 Jun 22;6(6):e19749. doi: 10.1371/journal.pone.0019749 (PMC3120814; doi:10.1371/journal.pone.0019749)
Supplement: Text S3 — HRMType Manual. (PDF) [file pone.0019749.s005.pdf]

HRMType®: a brief user's manual

Steven Tong

This is a brief description of how to use HRMType®, a program in STATA which allows the generation of typing schemes using a high resolution melt platform to interrogate amplified fragments.

The first section describes how to move the data from the MLST database into Stata in a way which allows Stata to use the full concatenated sequences.

1. Download the concatenated sequences from the MLST database as an Access file. Concatenate the whole sequence. Stata is unable to manage string variables of longer than 244, so cut the concatenated sequence up into 244 length fragments. Use the Mid([seq],1,244) command to do this. The name of each field should be named:
   st seq_1 seq_245 seq_489 seq_733 seq_977 seq_1221 seq_1465 seq_1709 seq_1953 seq_2197 seq_2441 seq_2685 seq_2929 seq_317
2. Cut and paste the table with the sequence types and corresponding 244 length sequences into Stata.
3. The HRMType do file generates the predicted curves. The main inputs are:
   a. Which sequence types you want to drop from the analysis.
   b. How many regions are to be interrogated.
   c. The start and end positions of these regions.

   The do file is named "generating_hrm_curve_list.do". An example is below

```
clear
set mem 80m
use "U:\infectious-diseases\HRMType\S_aureus\staph mlst in 244.dta", clear
destring st, replace

drop if length!=
drop if st==

local regions=5
local start1=150
local end1=300
local start2=1450
local end2=1600
local start3=457
local end3=620
local start4=2900
local end4=3050
local start5=2300
local end5=2530
```

Comment [ST1]: You can add or subtract which sequence types you want to exclude from the analysis.

Comment [ST2]: Choose how many regions to interrogate.

Comment [ST3]: Enter the start and end positions on the concatenated sequence of the region of interest. Note this is not where the primers start and end, but the amplified region inside of the primers.
Continue this for each of the regions of interest. To add more regions, just create further lines.

After running the file, the output you will see at the bottom of the screen will be the D value for your chosen regions. Further output can be obtained such as:

```
. list st b* MelT if st<21

      +--------------------------------------------+
      | st    b1      b2    b3    b4    b5    b6    MelT |
```

1.	1	53	24	13	43	66	43	202
2.	2	51	24.5	12	45	65	43	62
3.	3	53	24	13	43	66	43	202
4.	4	53	24.5	12	44	65	44	213
5.	5	53	23	12	43	66	43	174
6.	6	52	23	12	43	66	43	87
7.	7	52	23	12	44	65	43	91
8.	8	52	23	13	44	65	43	103
9.	9	52	23	13	43	66	43	100
10.	10	52	23	12	43	65	44	86
11.	11	53	23	12	43	66	43	174
12.	12	53	23	12	44	65	44	178
13.	13	53	23	12	44	65	44	178
14.	14	53	22	13	43	65	43	164
15.	15	54	22	13	43	65	43	237
16.	16	53	22	13	43	65	43	164
17.	17	51	23.5	12	44	65	42	34
18.	18	54	22	13	43	65	43	237
19.	19	51	24.5	12	44	62	43	55
20.	20	54	23	12	43	65	43	240

Steven Tong

Decmeber 2010
